# Typisierung von Floaterpatienten: Auswertung einer Studiendatenbank zum Zusammenhang von Merkmalen und Beeinträchtigung

**DOI:** 10.1007/s00347-020-01083-9

**Published:** 2020-03-27

**Authors:** Ursula Hahn, C. Baulig, S. Tulka, K. Ludwig

**Affiliations:** 1 OcuNet Verwaltungs GmbH, Friedrichstr. 47, 40217 Düsseldorf, Deutschland; 2 Fakultät für Gesundheit, Institut für Medizinische Biometrie und Epidemiologie (IMBE), Universität Witten-Herdecke, Witten-Herdecke, Deutschland; 3 ARIS Augenklinik, Nürnberg, Deutschland

**Keywords:** Floater, Soziodemographische Patientenmerkmale, Ophthalmologische Patientenmerkmale, Subjektiver Leidensdruck, Floaterspezifischer Fragebogen, Floaters, Sociodemographic patient characteristics, Ophthalmologic patient characteristics, Subjective suffering, Floater-specific questionnaire

## Abstract

Subjektive Beeinträchtigung von Floaterpatienten entzieht sich ophthalmologischen Messverfahren. Im Sinne einer Typisierung von Floaterpatienten sollen patientenbezogene Merkmale identifiziert werden, die mit höherer Beeinträchtigung assoziiert sind. Ein Datensatz einer prospektiven, multizentrischen, einarmigen Primärstudie zu 64 Floaterpatienten, die sich einer Vitrektomie unterzogen haben, umfasst neben patientenbezogenen Merkmalen Angaben zu subjektiver prä- und postoperativer (3 Monate nach Vitrektomie) Beeinträchtigung, die mit einem spezifischen Floaterfragebogen erhoben und zu relativen Beeinträchtigungsindizes (RBI) verdichtet wurden. Mediane der RBI wurden zu den Ausprägungen soziodemografischer und ophthalmologischer Parameter sowie der untersucherseitigen Beurteilung des Floaterausmaßes auf dem Studienauge und am zweiten Auge berechnet und auf Signifikanz getestet. Höhere präoperative RBI waren mit Berufstätigkeit, niedrigerem Alter, mit den präoperativen Befunden reduzierte Sehschärfe, Veränderungen der Netzhaut und höhere Fehlsichtigkeit sowie dem Vorliegen von Floatern am zweiten Auge verbunden. Höhere postoperative RBI lagen bei Indikation zur Kataraktoperation und niedrigerem Alter vor. Die RBI-Unterschiede waren nur punktuell signifikant. Die Behandlereinschätzung zum Ausmaß von Floatern einerseits und nach RBI andererseits waren nur für ansonsten augengesunde Patientenpopulationen signifikant korreliert. Die Primärstudie berichtete hohe präoperative Beeinträchtigung und erheblichen Rückgang der RBI nach Vitrektomie für nahezu alle Patienten. Die aktuelle Studie zeigt, dass einzelne patientenbezogene Merkmale tendenziell mit höherer präoperativer Beeinträchtigung und größerem Nutzen einer Vitrektomie verbunden sind.

## Abstract (en)

Subjective impairment of floater patients can only be partially quantified using ophthalmological test methods. In order to classify floater patients, patient-related characteristics associated with higher level of impairment should be identified. A data set of a prospective, multicenter single-arm primary study of 64 floater patients who underwent vitrectomy includes patient-related characteristics and information on subjective preoperative and postoperative (3 months after vitrectomy) impairment. Data on impairment were collected with a floater-specific questionnaire and condensed into self-rated impairment indices (SRI). Medians of the SRI were calculated for the manifestation of sociodemographic and ophthalmological parameters as well as for the classification of floaters on the study and second eye and tested for significance. Higher preoperative SRI were associated with professional activity, young age, reduced preoperative visual acuity, retinal alterations and higher refractive errors as well as floaters on the second eye. Higher postoperative SRI were found in combination with cataract and younger age. The SRI differences were only occasionally significant. Classification of floaters as documented by the surgeon and by SRI were significantly correlated only for patients without other visual comorbidities besides floaters. The primary study reported high preoperative subjective impairment and a significant decrease in SRI after vitrectomy for almost all patients. The current study showed that some individual patient-related characteristics tended to be associated with greater preoperative impairment and greater benefit from vitrectomy.

Das Ausmaß der subjektiven Beeinträchtigung von Floaterpatienten ist für Augenärzte nur bedingt nachvollziehbar. Daher sollen soziodemografische und ophthalmologische Merkmale identifiziert werden, die mit besonderer subjektiver Beeinträchtigung bzw. Gewinn infolge einer chirurgischen Therapie einhergehen. Die Typisierung soll den Augenarzt in der Beratungssituation unterstützen. Die subjektive Beeinträchtigung wurde mithilfe eines floaterspezifischen Fragebogens erfasst.

Geprüft wird zudem die Korrelation zwischen dem vom Untersucher befundeten Ausmaß der Floater und der subjektiven Beeinträchtigung.

## Einführung

Der Begriff „Floater“ beschreibt mobile Strukturen im Glaskörper, die so dicht sind, dass sie den Seheindruck beeinträchtigen können. Der Glaskörper des jungen Menschen besteht aus einer komplexen über Hyaluronsäure gebundenen Struktur, die zu 99 % aus Wassermolekülen besteht und durch ein Gerüst aus sehr dünnen Kollagenfasern organisiert wird [1]. Während des Alterungsprozesses nimmt der Anteil an Hyaluronsäure ab mit dem Ergebnis, dass Wassermoleküle freigesetzt werden und Kollagenfasern aggregieren. Mit zunehmender Dicke der Kollagenfasern und je nach ihrer Position zur Sehachse bzw. Nähe zur Netzhaut werden sie als flottierende Trübungen – daher auch der Name „Floater“ – wahrgenommen [2]. In einer späteren Phase löst sich der Glaskörper von der Netzhaut, in der Folge bildet sich bei einigen Menschen der sog: „Weiß-Ring“, ein besonders dicker Kollagenstrang, der sich nicht selten unmittelbar vor der zentralen Netzhaut befindet.

Zwei Behandlungsoptionen für Floater haben sich etabliert. Die chirurgische Pars-plana-Vitrektomie (PPV) und – seit kürzerer Zeit – die Vitreolyse mit einem kurz gepulsten Nd:YAG-Laser. Während die Evidenz und das Sicherheitsprofil für die kurz gepulste Nd:YAG-Laser-Behandlung noch nicht abschließend geklärt ist, ist die chirurgische Vitrektomie ein langjährig etabliertes Verfahren. Eine PPV entfernt Floater effektiv, die Operationsrisiken haben mit der Einführung minimal-invasiver Operationstechniken abgenommen. Induzierte Katarakt, iatrogene Netzhautforamina, Netzhautablösung und Endophthalmitis gehören aber zum Spektrum der Komplikationen [3–7].

Floater fallen den meisten Menschen kaum auf, eine geringe Anzahl von Betroffenen stören sie jedoch erheblich [7, 8]. Augenärzte sind bei der Empfehlung für eine Operation jedoch oft zurückhaltend. Dazu trägt neben der Abwägung des Komplikationsrisikos auch bei, dass das Krankheitsbild mit ophthalmologischen Messverfahren oft nicht objektivierbar und der Visus der Patienten – soweit keine ophthalmologischen Komorbiditäten vorliegen – häufig gut ist [7, 9]. In der Literatur wird zudem immer wieder berichtet, dass Ärzte auf Basis des Untersuchungsbefundes den subjektiven Leidensdruck von Patienten nicht oder nur partiell nachvollziehen können [6, 10].

Für die Untersuchung wird auf einen bereits vorhandenen Datensatz zurückgegriffen. Ziel der dem Datensatz zugrunde liegenden primären multizentrischen Studie war es, die subjektive funktionelle Beeinträchtigung über eine Kohorte von operationsbereiten Floaterpatienten mithilfe des modifizierten Visual Quality of Life Questionnaire (VQoL, [11]) über den Behandlungsverlauf (präoperativ, 3 und 12 Monate nach PPV) zu erfassen und als Indizes auszudrücken. Den Ergebnissen wurden die in der Studie erhobenen Komplikationen gegenübergestellt [12].

## Material und Methoden

### Eckdaten für den in der Auswertung verwendeten Datensatz

Der für die aktuelle Auswertung genutzte Datensatz basiert auf einer prospektiven multizentrischen einarmigen Beobachtungsstudie (Kohortenstudie ohne Vergleichskollektiv) ohne Verblindung [12]. Ziel dieser Studie war es, über eine Kohorte von Floaterpatienten die mit Floater assoziierte subjektive Beeinträchtigung präoperativ sowie die Änderung nach Vitrektomie 3 Monate sowie 12 Monate postoperativ zu erheben. Die Vitrektomie erfolgte ausschließlich aufgrund von Floatern. Der Nutzen im Sinne reduzierter Beeinträchtigung wurde ins Verhältnis zu den chirurgischen Risiken der Vitrektomie bewertet.

Eingeschlossen wurde jeweils 1 Auge von Patienten, die mindestens 18 Jahre alt waren und in der OCT eine intakte Makula aufwiesen. Ausgeschlossen wurden Patienten mit anderen Vorerkrankungen wie deutliche Katarakt, Hornhauttrübung oder Glaskörpertrübung, die mit einem Visus ≤0,63 vergesellschaftet waren, sowie einem intraokularen Eingriff am zu operierenden Auge innerhalb der letzten 6 Monate. Die Vitrektomie wurde als 3‑Port-PPV in 23-G-Technik mit Entfernung des Glaskörpers bis zum Äquator durchgeführt. Im gleichen Eingriff und bis einschließlich der 3‑Monats-Nachkontrolle waren weder eine Kataraktoperation noch eine Nachstarentfernung zugelassen, dies hätte zum Ausschluss aus der Studie geführt.

Zwei der insgesamt 6 Studientermine lagen präoperativ: Die Patienten sollten nach intensiver Aufklärung zunächst in Ruhe überlegen können, ob sie den elektiven Eingriff umsetzen wollten. Der formale Einschluss der Patienten erfolgte spätestens 1 bis 2 Tage vor der Operation, die postoperativen Termine fanden jeweils 1 bis 2 Tage, 10 bis 14 Wochen (kurz 3 Monate) und 10 bis 14 Monate (kurz 1 Jahr) nach dem Eingriff statt. Zu jedem Studientermin wurden ophthalmologische Parameter (Visus cc, Sphäre, Zylinder, Spaltlampenbefund, Fundusbefund in Mydriasis) erfasst. Die Untersucher waren beim 2. präoperativen Termin aufgefordert, das Ausmaß der Floater auf dem Studienauge (SA) und am zweiten Auge (ZA) entlang der Skala „kein Floater“ (nur zweites Auge), „gering“, „mäßig“, „erheblich“, „massiv“ zu klassifizieren. Präoperativ wurden soziodemografische Angaben der Studienpatienten (Alter, Geschlecht, Berufstätigkeit) erfasst. Studienpatienten wurden gebeten, den immer gleichen floaterspezifischen Fragebogen zur Beeinträchtigung am 2. präoperativen sowie zum 2. und 3. postoperativen Termin auszufüllen.

Die Studie wurde nach „good clinical practice“ durchgeführt; neben dem Votum der Kopfethikkommission Bayern (Nr. 11061 vom 11.08.2011) wurden auch die für die regionalen Studienzentren zuständigen Ethikkommissionen einbezogen. Studienkoordination, Datamanagement und Monitoring lagen beim OcuNet Verbund, Düsseldorf. Die Fallzahlplanung erfolgte auf Basis einer Pilotstudie, für die Details verweisen wir auf die Veröffentlichung zur Primärstudie [12]. Die Fallzahlplanung und Auswertung wurden in Kooperation mit dem Institut für medizinische Biometrie und Epidemiologie (IMBE) der Universität Witten/Herdecke umgesetzt, die Veröffentlichung der Ergebnisse erfolgte im Jahr 2018 [12].

Der Fragebogen zur Quantifizierung der subjektiven funktionellen Beeinträchtigung bei Floatern zu verschiedenen Sehqualitäten basiert partiell auf dem „Visual Quality of Life“-Fragebogen (VQoL) [11]. Aus dem VQoL wurden 9 Einzelfragen herangezogen, die um 5 Fragen zu den bei Floatern typischen beweglichen Trübungen ergänzt wurden, Details zu den Vorarbeiten zur Gestaltung des Fragebogens sind in der Veröffentlichung zur Primärerhebung dargestellt [12]. Der in der Datenerhebung verwendete Fragebogen greift die Antwortskala des VQoL auf: Von den jeweils 6 Kategorien (deren Ausformulierung fragenspezifisch abweichen kann), erfasst der kleinste Wert („0“) keine Beeinträchtigung und der höchste Wert („5“) die größte Beeinträchtigung.^1^

Zur weiteren Auswertung wurden die Ordinalwerte der Antwortkategorien in stetige Indizes umgerechnet. Dazu wurde über die Einzelfragen (mit vorliegender Antwort) die Summe der Antwortkategorien ermittelt und in einen relativen Beeinträchtigungsindex (RBI) mit dem Wertebereich 0–100 % transformiert. Ein RBI von 0 % steht für keine, ein RBI von 100 % für maximale Beeinträchtigung.

„RBI gesamt“ bezieht sich auf alle Einzelfragen, für jeweils unterschiedliche Sehqualität wurden Subindizes aus einer Auswahl von Einzelfragen ermittelt:

- „RBI1 allgemein“ umfasst die Einzelfragen zum Gesamteindruck Sehqualität (getrennt nach Augen, in die Auswertung wurde nur die Frage zum Studienauge einbezogen) und Angst vor Verschlechterung,
- „RBI2 Blendung“ die Einzelfragen, die sich nach Blendung aufgrund von Kunstlicht und Sonnenlicht erkundigen,
- „RBI3 Nahsicht“ die Einzelfragen, mit denen nach Einschränkungen bzw. Beeinträchtigung beim Lesen generell bzw. Lesen kleiner Schrift gefragt wird, und
- „RBI4 bewegliche Trübungen“ die Einzelfragen, mit denen nach Problemen aufgrund beweglicher Punkte, Wolken, Flecken oder Fäden gefragt wird.

Um die Entwicklung über den Untersuchungsverlauf sichtbar zu machen, wurde intraindividuell für jeden Patienten die absolute Differenz zwischen relativem Beeinträchtigungsindex zum 2. präoperativer Termin (Baseline) und zum 2. bzw. 3. postoperativen Termin erhoben und die relative Veränderung in Prozent berechnet. Negative Werte stehen für eine Abnahme, positive für eine Verstärkung der gemessenen Beeinträchtigung. Zu weiteren Details der Methodik aus der Primärstudie verweisen wir auf die Veröffentlichung [12].

Zwischen 2012 und 2016 wurden 69 Patienten aus 8 Studienzentren eingeschlossen, davon wurden schlussendlich 64 Patienten operiert; 62 Patienten nahmen den Termin nach 3 Monaten und 59 nach 12 Monaten wahr.

### Fragestellung der aktuellen Analyse und statistische Auswertung

Die Daten der Primärstudie wurden für 2 Fragestellungen herangezogen. Zum einen sollen im Sinne einer Typisierung von Floaterpatienten soziodemografische und ophthalmologische Merkmale identifiziert werden, die mit höherer subjektiver Beeinträchtigung einhergehen. Zum anderen sollte die Ausprägung der Floater nach Einschätzung durch den jeweiligen Untersucher auf Korrelation mit der subjektiven Beeinträchtigung überprüft werden.

Zur Typisierung von Floaterpatienten nach Merkmalen wurden die Mediane der relativen Beeinträchtigungsindizes (RBI) jeweils getrennt nach Merkmalsausprägung (also z. B. für Männer und Frauen) ermittelt. Die Analyse umfasste die soziodemografischen Merkmale Geschlecht, Alter (gruppiert in folgende Altersgruppen: 40 bis 49, 50 bis 59, 60 bis 69 und 70 bis 79) und Berufstätigkeit. Zu den ausgewerteten ophthalmologischen Merkmalen zählten prä- und postoperativer Visus cc des operierten Auges binarisiert zu Visus cc <0,8 und Visus cc ≥0,8, Fehlsichtigkeit binarisiert zu sphärischen Äquivalenten ≤|2 dpt| zu >|2 dpt|, präoperatives Vorliegen von perimakulären und peripheren Veränderungen sowie Indikation zur Kataraktoperation 3 Monate nach der Vitrektomie. Zudem wurden die RBI-Mediane nach dem präoperativen Ausmaß der Floater auf dem Studienauge sowie Vorliegen/Nicht-Vorliegen von Floatern am zweiten Auge (jeweils nach Einschätzung des Untersuchers) ausgewertet.

Die Mediane der präoperativen RBI gesamt (im Folgenden „präop RBI“) sowie postoperativen RBI gesamt (im Folgenden „postop RBI“) wurden für die zu prüfenden Merkmale univariat stratifiziert in 3 Abbildungen (soziodemografische Merkmale, ophthalmologische Merkmale, klinische Bewertung von Floatern auf dem Studienauge und am zweiten Auge) als Streudiagramme abgetragen. In der Abszisse (x-Achse) ist der präop RBI abgetragen; je höher der Wert, umso ausgeprägter erlebte der Studienpatient die präoperative Beeinträchtigung. In der Ordinate (y-Achse) ist der postop RBI nach 3 Monaten abgetragen; je niedriger dieser Wert, umso geringer war die subjektive postoperative Beeinträchtigung. Der gleichzeitige Abtrag des präop und postop RBI visualisiert sowohl die präoperative als auch die postoperative subjektive Beeinträchtigung und erlaubt damit einen Rückschluss auf den Nutzen der Vitrektomie: So stehen ein hoher präop und ein niedriger postop RBI (unterer rechter Quadrant) für einen hohen Rückgang einer ursprünglich hohen subjektiven Beeinträchtigung und repräsentieren damit einen hohen Gewinn aus der Vitrektomie. Hingegen lässt ein präop und postop niedriger RBI (unterer linker Quadrant) einen vergleichsweise geringen Rückgang der subjektiven Beeinträchtigung erkennen.

Zur Evaluation des Einflusses verschiedener Sehqualitäten wurden ergänzend merkmalsspezifisch die Mediane zu den Subindizes RBI1 „Allgemein“, RBI2 „Blendung“, RBI3 „Nahsicht“ und RBI4 „bewegliche Trübungen“ aus den präoperativen Angaben berechnet. Die merkmalsbezogenen Mediane der RBI gesamt sowie nach Subindizes wurden auf lokale Signifikanz getestet. Je nach Datenqualität wurde das Vorliegen eines lokal statistisch signifikanten Unterschieds zwischen den beiden Gruppen mithilfe von zweiseitigem Wilcoxon- (stetige Daten) bzw. dem Exakten Fisher-Text (kategoriale Daten) geprüft. Die Merkmale der 3 Merkmalsgruppen – soziodemografische und ophthalmologische Merkmale sowie untersucherseitige Einschätzung des Ausmaßes von Floatern auf dem Studien- und am zweiten Auge – wurden dazu jeweils zum präop RBI auf Signifikanz getestet. Eine signifikante Assoziation wurde bei einem *p*-Wert ≤0,05 angenommen. Die Ergebnisse der Analyse nach Subindizes und der Signifikanzprüfung werden im Ergebnisteil ohne grafische oder tabellarische Unterlegung vorgestellt. Der abschließende Analyseschritt basiert auf einer multivariaten linearen Regression. Die Merkmale der 3 Merkmalsgruppen – soziodemografische und ophthalmologische Merkmale sowie untersucherseitige Einschätzung des Ausmaßes von Floatern auf dem zweiten Auge – wurden dazu jeweils zur Zielgröße präop RBI auf Signifikanz getestet.

Zur Überprüfung der Übereinstimmung von berichteter subjektiver Beeinträchtigung der Probanden einerseits und der Klassifikation des Behandlers zum Ausmaß der Floater auf dem Studienauge andererseits wurden die Mediane des präoperativen RBI gesamt getrennt nach der vom Behandler gewählten Ausmaßklasse ausgewiesen. Davon ausgehend, dass andere ophthalmologische Komorbiditäten als Floater die Bewertung der Probanden überlagern könnten, erfolgte diese Auswertung nicht nur in der gesamten Studienpopulation, sondern auch in der Teilkohorte mit gutem Visus und ohne ophthalmologische Komorbiditäten (keine Linsentrübung/Kapselsacktrübung, keine Veränderungen der Netzhaut). Die Prüfung auf einen lokal statistisch signifikanten Unterschied erfolgte für beide Populationen über den Kruskal-Wallis-Test. Eine lokal signifikante Assoziation wurde bei einem *p*-Wert ≤0,05 angenommen.

Die Prüfung auf einen lokal statistisch signifikanten Unterschied für beide Populationen erfolgte über den Kruskal-Wallis-Test.

## Ergebnisse

### Soziodemografische und ophthalmologische Merkmale

Die Verteilung soziodemografischer und ophthalmologischer Merkmale sowie die Untersuchereinschätzung zum Ausmaß der Floater auf Studienaugen und zweiten Augen sind in Tab. 1 wiedergegeben. Der Frauenanteil überwog, die Patienten waren im Median 64 Jahre alt, rund ein Drittel war berufstätig; 81 % der Studienpatienten wiesen einen Visus cc von mindestens 0,8 auf, der Anteil der Patienten mit hohen Fehlsichtigkeiten (sphärisches Äquivalent >|2 dpt|) betrug 32 %. Bei 51 % der Patienten diagnostizierten die Behandler auch am zweiten Auge Floater. Kein Studienauge wies eine Pathologie an der Hornhaut auf. Veränderungen der Netzhaut wurden bei 13 Patienten (19 %) beschrieben, 3 davon betrafen perimakuläre Veränderungen, 10 nur oder auch die Peripherie.

**Table 1:** 

	*N*	In %
Alle	69	–
Rechtes Auge	36	52,2
Linkes Auge	33	47,8
Geschlecht		
Männlich	30	43,5
Weiblich	39	56,5
Alter		
40–49	10	14,5
50–59	12	17,4
60–69	26	37,7
70–79	20	29
≥80	1	1,4
Patienten berufstätig		
Ja	26	37,7
Nein	43	62,3
Präoperativer Visus cc des operierten Auges		
Visus cc < 0,8	13	19,4
Visus cc ≥ 0,8	54	80,6
Präoperatives sphärisches Äquivalent des operierten Auges		
≥−2 und ≤2 dpt incl	45	68,2
<−2 und >+2 dpt	21	31,8
Untersuchereinschätzung: Floater auf dem Studienauge		
Gering	2	3,2
Mäßig	20	31,7
Erheblich	34	54
Massiv	7	11,1
Untersuchereinschätzung: Floater am zweiten Auge		
Kein Floater	31	49,2
Gering	8	12,7
Mäßig	15	23,8
Erheblich	7	11,1
Massiv	2	3,2

### Typisierung von Floaterpatienten

**Figure 1:**
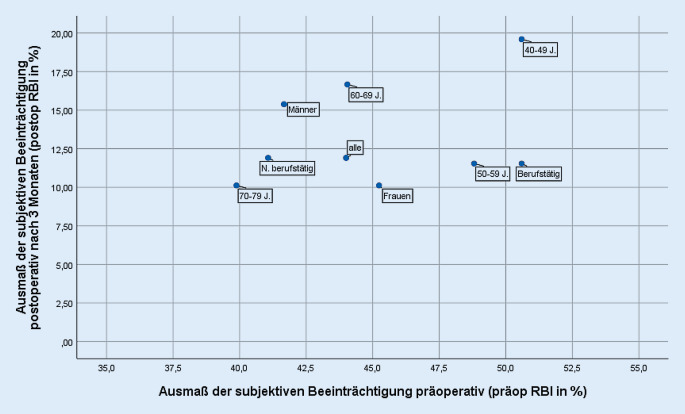

Über alle Floaterpatienten der Primärstudie lag der präop RBI bei 44 % und der postop RBI (3 Monate nach Vitrektomie) bei 12 % (Abb. 1, 2 und 3). Merkmalsspezifisch wiesen die beiden Kenngrößen zum Teil erhebliche Unterschiede auf.

**Figure 2:**
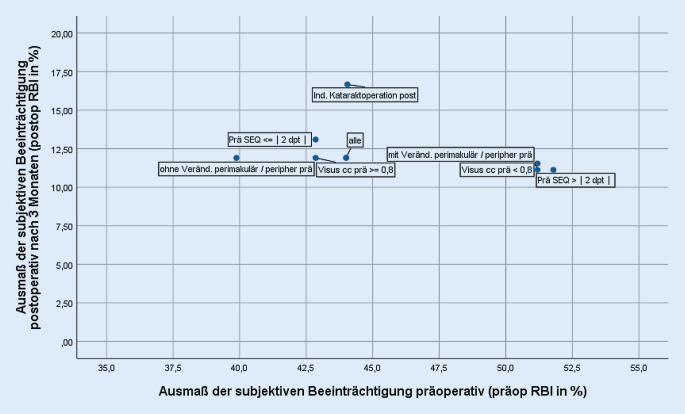

**Figure 3:**
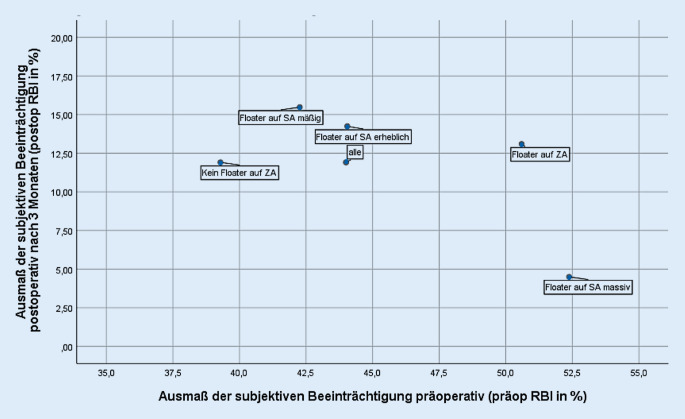

Frauen profitierten stärker von der chirurgischen Therapie: Sie berichteten präoperativ über einen höheren RBI als Männer (präop RBI Männer: 42 %, Frauen: 45 %) und postoperativ über einen um 5 Prozentpunkte niedrigeren (postop RBI Männer: 15 %, Frauen: 10 %) (Abb. 1). Die RBI-Verteilung und Entwicklung nach Altersgruppen ist uneinheitlich: Studienpatienten in den mittleren Altersgruppen wiesen bei mittlerem präop RBI (50 bis 59 Jahre: 49 %, 60 bis 69 Jahre: 44 %) die höchsten Rückgänge in der Beeinträchtigung auf (postop RBI: 12 %, 17 %), junge Probanden berichteten einen etwas höheren präop RBI (40 bis 49 Jahre: 51 %), der postop RBI lag jedoch über dem der älteren Probanden (20 %). Ältere Studienteilnehmer berichteten vergleichsweise niedrige präop und postop RBI (70 bis 79 Jahre: präop RBI 40 %, postop RBI 10 %). Weder zu Geschlecht noch zu Alter waren die Assoziation für präop oder postop RBI oder für die Subindizes RBI1 bis RBI4 zum 5 %-Niveau lokal signifikant.

Unter allen soziodemografischen Merkmalen zeigten sich die größten Unterschiede zum Merkmal Berufstätigkeit. Berufstätige gaben höhere präop RBI (51 % zu 41 % bei nichtberufstätigen Floaterpatienten) an. Da die postop RBI für beide Subgruppen das gleiche Niveau erreichten (Berufstätige und Nichtberufstätige 12 %), realisierten die Berufstätigen einen deutlich höheren Rückgang der Beeinträchtigungswerte und damit einen höheren Nutzen aus der Vitrektomie. Die Auswertung nach Subindizes (RBI1 bis RBI4) lässt erkennen, dass sich Berufstätige präoperativ insbesondere in der Nahsicht (RBI3) und aufgrund beweglicher Trübungen (RBI4) beeinträchtigt fühlten (RBI3: Berufstätige 58 %, Nichtberufstätige 50 %; RBI4 57 % zu 37 %), zum 5 %-Niveau ist nur die Abweichung zum RBI4 signifikant.

Patienten mit vorbestehenden Veränderungen an der Netzhaut (perimakulär und peripher) berichteten präoperativ höhere RBI als die nicht belastete Vergleichsgruppe (51 % mit zu 40 % ohne Veränderungen), nicht jedoch postoperativ (Abb. 2). In der Auswertung nach Subindizes (RBI1 bis RBI4) ließen sich zwischen den Subgruppen deutliche Unterschiede für die Sehqualitäten Blendung (RBI2) und bewegliche Trübungen (RBI4) zeigen (RBI2: mit Veränderungen 61 %, ohne 44 %; RBI4 53 % zu 43 %). Die Abweichung zum RBI2 war zum Signifikanzniveau von 5 % lokal statistisch signifikant.

Patienten mit niedrigerem präoperativem Visus cc berichteten höhere präop RBI (51 % bei Patienten mit Visus cc < 0,8 zu 43 % bei Patienten mit Visus cc ≥ 0,8), erreichten aber postoperativ das RBI-Niveau der Vergleichsgruppe. Das Muster nach einzelnen Sehqualitäten (RBI1 bis RBI4) entsprach dem bei Veränderungen der Netzhaut: Patienten mit niedrigerem Visus cc fühlten sich präoperativ überproportional durch Blendung (RBI2 61 % zu 44 %) und bewegliche Trübungen (RBI4 57 % zu 43 %) gehandicapt. Weder zum präop noch zum postop RBI oder den Subindizes ließen sich lokal signifikante Assoziation zeigen.

Der postoperative Visus cc hatte keinen Einfluss auf die Höhe des postop RBI. Auffällig ist lediglich die Subgruppe, die zum 3‑Monats-Untersuchungstermin eine Indikation zur Kataraktoperation aufwies: Der postop RBI war höher als der von Patienten ohne Indikation (mit Indikation: 17 %, ohne Indikation 12 %).

Patienten mit höheren Fehlsichtigkeiten wiesen einen höheren präop RBI auf (52 % bei Patienten mit sphärischem Äquivalent >|2 dpt| zu 43 % bei Patienten mit keiner/niedrigerer Fehlsichtigkeit), die postop RBI der beiden Subpopulationen lagen hingegen dicht beieinander (11 % zu 13 %). Nach Sehqualitäten wurden die größten Abweichungen zu Blendung (RB2: 67 % zu 44 %) und zu beweglichen Trübungen (RB4 53 % zu 43 %) realisiert. Nur die Abweichung zum RBI2 war zum 5 %-Niveau lokal signifikant.

Lag laut Untersucherbefund kein Floater am zweiten Auge vor, waren die präop RBI niedriger als bei diagnostizierten Floatern (39 % ohne Floater, 51 % mit Floater am zweiten Auge), die postop RBI in den beiden Subgruppen wichen jedoch kaum voneinander ab (12 % zu 13 %) (Abb. 3). In den Subgruppen, die laut Untersucherbefund auf dem Studienauge Floater „mäßiger“ oder „erheblicher“ Ausprägungen aufwiesen, lagen die präop und postop RBI-Werte dicht beieinander. Anders verhält es sich für die Patientenkohorte mit „massiver“ Ausprägung von Floatern: Der präop RBI war deutlich höher (rund 10 Prozentpunkte) und der postop RBI deutlich niedriger als in den anderen Klassen. Das diagnostizierte Vorliegen von Floatern am zweiten Auge war zum präop RBI (*p* = 0,033) und dem Subindex RBI2 (Blendung) (*p* = 0,005) zum 5 %-Niveau signifikant.

### Ergebnisse der Regressionsanalyse

In linearen Regressionsanalysen wurde der Zusammenhang der 3 Merkmalsgruppen (soziodemografische und ophthalmologische Merkmale sowie Untersuchereinschätzung) zum präop RBI (abhängige Variable) getestet. Die Tab. 2 verdeutlicht die Ergebnisse der multivariaten linearen Regressionen für die Zielvariable präop RBI zu den präoperativen soziodemografischen und ophthalmologischen Merkmalen der Studienpatienten. In das Modell wurden mittels Vorwärtsselektion die präoperativen Variablen Alter, Geschlecht, Berufstätigkeit, Visus, sphärisches Äquivalent und Veränderungen der Netzhaut aufgenommen. Niedrigeres Alter, Geschlecht (Männer), guter Visus cc (≥0,8), geringes sphärisches Äquivalent (≤|2 dpt|) führten zu einem verringerten Wert der Zielvariable präop RBI, während Vorliegen von Berufstätigkeit, von Veränderungen an der Netzhaut und von Floater auf dem zweiten Auge zu einer Erhöhung führten. Alle Einflüsse waren zu einem Signifikanzniveau von 5 % nicht lokal signifikant.

**Table 2:** 

Präoperative Variable	Effektschätzer	95 %-Konfidenzintervall	*p*-Wert
Konstante	52,0	28,1–75,9	0,00
Alter (gruppiert in folgende Altersgruppen: 40–49, 50–59, 60–69, 70–79)	−1,9	−7,7–3,9	0,52
Geschlecht des Patienten	−3,7	−11,6–4,2	0,35
Berufstätigkeit	2,7	−9,9–15,2	0,67
Visus cc (binarisiert zu ≥0,8 und <0,8)	−4,3	−15,2–6,6	0,43
Sphärisches Äquivalent (binarisiert ≤|2 dpt| zu >|2 dpt|)	−4,9	−14,9–5,2	0,33
Veränderungen der Netzhaut außerhalb der Makula	4,2	−6,6–15,0	0,44
Vorliegen ein Floater auf dem zweiten Auge	7,4	−1,4–16,1	0,10

### Übereinstimmung der Befundung des Behandlers und des subjektiven Leidensdrucks?

Die untersucherseitige Klassifikation zum Ausmaß von Floatern auf dem Studienauge und die präop RBI über die gesamte Studienpopulation zeigten zwar einen parallelen Verlauf – je größer das Ausmaß der Floater laut Untersuchereinschätzung, umso höher ist der präop RBI (Tab. 3). Wie schon in der Primärstudie gezeigt wurde [12], lagen die präop RBI jedoch für die Untersucherklassifikationen „gering“, „mäßig“ und „erheblich“ sehr dicht beieinander. In der jetzt zusätzlich erfolgten Analyse für die Subpopulation der mit Ausnahme von Floatern augengesunden Studienteilnehmer zeigten sich analoge Verhältnisse der Lagemaße von präop RBI und Untersuchereinschätzung. Anders als in der gesamten Studienpopulation wies nur diese Subpopulation eine Assoziation zum 5 %-Niveau auf. Das bestätigt sich auch nach Subindizes (RBI1 bis RBI4): Nur in der Subpopulation der augengesunden Patienten war die Assoziation, bezogen auf die Sehqualitäten Nahsicht (RBI3) und bewegliche Trübungen (RBI4), lokal signifikant (RBI3: *p* = 0,003, RBI4: *p* = 0,026).

**Table 3:** 

		Population gesamt		Population mit präop. Visus ≥0,8, keine Trübung, keine Veränderungen der Netzhaut	
		*N*	RBI gesamt, Median	*N*	RBI gesamt, Median
Untersucherseitige Klassifikation des Ausmaßes von Floatern auf dem zu operierenden Auge	Gering	2	–	1	–
	Mäßig	18	42 %	11	38 %
	Erheblich	33	44 %	15	39 %
	Massiv	7	52 %	3	71 %
		–	*p* = 0,490	–	*p* = 0,019

*RBI* relativer Beeinträchtigungsindex

## Diskussion

Floater sind ein häufiges Phänomen in der augenärztlichen Beratung, selbst junge Menschen geben mit Floatern assoziierte Symptome an [13]. Floater treten überproportional häufig bei Myopien und Hyperopien auf [2, 14], sie kommen jedoch unabhängig von Fehlsichtigkeiten, Alter und Geschlecht vor. Patienten stellen sich in der augenärztlichen Praxis oft mit der Frage nach einer Therapie vor. „Nach Aufklärung der Patienten über die Harmlosigkeit des Befundes ist die Mehrzahl der Patienten beruhigt und kann damit leben“ [7]. Während sich viele an das Phänomen Floater „gewöhnen“, fehlt die Adaptationsfähigkeit bei anderen [1, 15]. Es bleibt damit eine Restgruppe an Patienten, die eine Therapie trotz der damit verbundenen Risiken wünscht [13, 16, 17].

Das stellt Augenärzte vor ein Dilemma: Das Krankheitsbild ist mit der üblichen ophthalmologischen Messmethodik nicht gut zu erfassen, und die Operation ist mit Risiken verbunden. Nicht immer ist die von Patienten angegebene Beeinträchtigung aufgrund des ophthalmologischen Untersuchungsbefunds nachvollziehbar [6, 10]. Dass Floaterpatienten als schwierige Patienten gelten, könnte in der speziellen Arzt-Patienten-Interaktion begründet sein: Patienten haben das Gefühl, mit ihrem Anliegen nicht ausreichend ernst genommen zu werden. Die ärztliche Einschätzung, dass es sich bei Floatern nicht um eine behandlungsbedürftige Pathologie handelt, ist für diese Patienten frustrierend. „From their point of view, the consulting ophthalmologist who sought evidence of disease and found none has nonetheless failed to address their health and quality-of-life issues.“ [17]

Verschiedene Quellen beschreiben Floaterpatienten mit hohem Leidensdruck als sehr differenziert, anspruchsvoll, psychisch belastet und mit höheren Prävalenzen psychiatrischer Diagnosen als die Kontrollgruppen [7–9, 18]. Dieser Besonderheit des Patientenkollektivs muss in der Beratungssituation Rechnung getragen werden.

Subjektives Erleben des Nutzens von Vitrektomie bei Floatern war bereits Gegenstand verschiedener Studien. Dabei standen die globale individuelle Zufriedenheit und der patientenorientierte Nutzen im Vordergrund. Die berichtete Zufriedenheit und der Nutzen der Operationen waren in den betrachteten Populationen durchgängig hoch [5–7, 17, 19]. Bislang fehlen jedoch Studien mit systematischer Untersuchung, welche soziodemografischen und ophthalmologischen Merkmale von Patienten mit der subjektiven Beeinträchtigung von Patienten in Wechselwirkung stehen. In einigen Veröffentlichungen wird dieser Aspekt zwar auf Basis der Ergebnisse mit diskutiert, steht aber nicht im Zentrum der Untersuchungen. Zudem sind die Ergebnisse uneinheitlich.

In der Literatur besteht Einigkeit, dass die Operation bei der richtigen Patientenauswahl zur Qualität des Sehens und zur Patientenzufriedenheit beiträgt [15]. Um die „richtigen“ Patienten für eine Operation zu identifizieren, haben wir Floaterpatienten, die sich einer PPV unterzogen haben, unter dem Gesichtspunkt typisiert, welche soziodemografischen und ophthalmologischen Merkmale mit höherer subjektiver Beeinträchtigung einhergehen. Ziel der Auswertung ist es, dem beratenden Augenarzt Hilfestellung für die Beratungssituation an die Hand zu geben. Liegen bei dem Patienten Merkmale vor, die nach den Ergebnissen dieser Auswertung mit hoher subjektiver präoperativer Beeinträchtigung und/oder einem hohen Rückgang der Beeinträchtigung assoziiert sind, dann kann eine Operation auch dann zu einer Besserung führen, wenn der behandelnde Augenarzt dies aufgrund der ophthalmologischen Untersuchungswerte nicht unmittelbar nachvollziehen kann.

Diese Auswertung dient zudem zur Überprüfung der These, dass Augenärzte die funktionelle Beeinträchtigung auf Basis von qualitativen Untersuchungsbefunden nicht reproduzieren können. Dazu wurde der subjektive Beeinträchtigungsindex der Klassifikation zum Ausmaß der Floater in verschiedenen Patientenpopulationen gegenübergestellt.

### Rationale und Ergebnisse der Primärerhebung

Der Datensatz für die aktuelle Auswertung entstammt einer Primärstudie, in der die Entwicklung der subjektiven Beeinträchtigung operationsbereiter Patienten mithilfe eines floaterspezifischen Fragebogens über den Behandlungsverlauf mit Vitrektomie evaluiert wurde. Die Frage lautete, wie groß der Nutzen im Sinne von verminderter Beeinträchtigung nach Vitrektomie in der Kohorte für sich betrachtet und im Verhältnis zu den Operationsrisiken ist. Die vorliegende Auswertung fokussiert hingegen auf Patientenmerkmale. Sie prüft, welche Patientencharakteristiken mit höherer oder niedrigerer präoperativer Beeinträchtigung bzw. mit höherem oder niedrigerem Nutzen aus der Vitrektomie einhergehen.

Anders als in dieser Auswertung erfolgte keine nach Patientenmerkmalen differenzierende Analyse [12]. Über die gesamte Kohorte berichteten Studienpatienten von erheblicher präoperativer Beeinträchtigung, die über den Behandlungsverlauf bei nahezu allen erheblich zurückging. Der RBI lag präoperativ bei 44 %, 3 Monate nach PPV bei 12 % (s. auch Abb. 1, 2 und 3). Bei 8 Patienten traten in der Primärstudie intra- oder postoperativ Komplikationen auf, das entspricht den in der Literatur berichteten Inzidenzen [12]. Komplikationen hatten keinen negativen Effekt auf die in dieser Studie gemessene subjektive Beeinträchtigung: Im Gegenteil lag in der Kohorte der Patienten mit Komplikationen die postop RBI gesamt zum 12-Monats-Zeitpunkt noch niedriger als in der Vergleichsgruppe. Für die bei nahezu allen Studienpatienten gemessene postoperative Restbeeinträchtigung wurden in der Primärveröffentlichung verschiedene Gründe, wie z. B. persistierende Floater auf dem zweiten Auge, keine vollständige Glaskörperentfernung bis in die Glasköperbasis, andere visusrelevante Pathologien, diskutiert [12].

### Patientenbezogene Merkmale und subjektive Beeinträchtigung

Dass die weit überwiegende Mehrzahl der operationsbereiten Studienpatienten deutlich von der Vitrektomie profitierte, ist das zentrale Ergebnis der Primärstudie. Mit dieser Auswertung ließen sich einige Trends ableiten, welche soziodemografischen und ophthalmologischen Merkmale mit höherer präoperativer subjektiver Beeinträchtigung oder hohen Reduktionen der Beeinträchtigung über den Behandlungsverlauf vergesellschaftet sind. Insgesamt sind die Zusammenhänge aber eher schwach, lokale statistische Signifikanz lässt sich nur punktuell zeigen. Eine Studie, die den Parameter „Nutzen“ verwendete, berichtet ebenfalls Trends, sieht aber gar keine signifikanten Zusammenhänge [19].

In dieser Auswertung ist der wichtigste soziodemografische Faktor mit Einfluss auf das präoperative Maß der Beeinträchtigung (präop RBI) die Berufstätigkeit (Abb. 1). Im Berufsleben stehende Personen erlebten Floater als beeinträchtigender und berichteten einen höheren Nutzen der Vitrektomie als nichtberufstätige Personen (lokal signifikant zum präop RBI). Die Beeinträchtigung wurde zu bestimmten Sehqualitäten, die bei Berufstätigkeit stärker gefordert sind, besonders deutlich: rasches Einschätzen einer Situation bzw. gutes Erkennen von Gesichtern von Gesprächspartnern kann durch den beweglichen Charakter der Floater behindert sein (RBI4), Probleme in der Nahsicht (RBI2) erschweren das Lesen. Die Vermutung, dass berufliche Anforderungen mit einem höheren Leidensdruck assoziiert sind, wurde auch bereits anderweitig in der Literatur formuliert [5, 7, 8]. Stoffelns et al. bestätigen diesen Befund indirekt. In einer Veröffentlichung aus 2011 arbeiten die Autoren heraus, dass akademische Berufe und Berufe, für die Lesen eine Voraussetzung ist (konkret Lehrer, Rechtsanwälte, Geschäftsleute und Universitätsabgänger), bei operationswilligen Floaterpatienten überdurchschnittlich hoch sind [8].

Das Alter des Patienten ist in verschiedener Hinsicht für eine Indikationsstellung zu einer Vitrektomie relevant: Die älteste Kohorte der 70- bis 79-Jährigen berichtete vergleichsweise niedrigere präoperative wie postoperative Beeinträchtigungswerte (Abb. 1). Die primär geringere subjektive Beeinträchtigung und der geringere Nutzen einer Vitrektomie lassen sich auch für nicht (mehr) im Erwerbsleben Stehende zeigen. Der Nutzen für diese Patientengruppen ist relativ geringer als bei den Vergleichskohorten, absolut profitieren sie jedoch auch von der Vitrektomie. Der präop RBI der 70- bis 79-Jährigen liegt bei 40 % und damit 30 Prozentpunkte höher als der postop RBI (10 %). In der Beratung ist jedoch zu erwägen, Patienten, die kurz vor Berufsende stehen, ein Abwarten bis nach Beginn des Ruhestands vorzuschlagen. Es ist denkbar, dass Floater für Patienten in dieser Lebensphase erträglicher werden. Das Merkmal geringes Alter sollte Anlass für eine (zusätzliche) kritische Überprüfung der Operationsindikation sein: Die Patienten der Alterskohorte 40 bis 49 Jahre berichteten zwar über eine hohe subjektive Ausgangsbeeinträchtigung, sie profitierten auch von der Vitrektomie, im Vergleich zu anderen Altersgruppen ist jedoch der postoperative RBI in dieser Kohorte höher. Ein hoher subjektiver Leidensdruck jüngerer Patienten und eine höhere Bereitschaft, für eine Therapie Risiko auf sich zu nehmen, wird auch anderweitig berichtet [19]. Denkbar ist, dass die berichteten (subjektiven) Beeinträchtigungen ursächlich (auch) auf die beginnende Altersweitsichtigkeit zurückzuführen sind.

Höhere präoperative Fehlsichtigkeit, schlechterer Ausgangsvisus und Vorliegen von perimakulären und peripheren Veränderungen der Netzhaut sind die ophthalmologischen Merkmale, die mit höherem präop RBI einhergehen (Abb. 2), das Niveau der postop RBI entspricht dem der nicht belasteten Vergleichsgruppen. Es scheint so zu sein, dass Patienten dieser Subgruppen ihre Floater als besonders störend empfinden und insofern von einer Vitrektomie überdurchschnittlich profitieren könnten. Dass Patienten mit schlechterem Ausgangsvisus (jedoch nicht schlechter als 0,6 cc) nur präoperativ auch erhöhte RBI aufwiesen, postoperativ jedoch nicht mehr, spricht zumindest dafür, dass eine moderat verminderte Sehschärfe offenbar die subjektive Beeinträchtigung durch Floater nicht verringert, mit anderen Worten, dass eine moderat verminderte Sehschärfe nicht vor Beeinträchtigung durch Floater schützt. Dass der RBI in dieser Subgruppe postoperativ zu dem der unbelasteten Vergleichsgruppe aufschloss, obwohl durch die Vitrektomie nur die Floater entfernt wurden, spricht für einen Beitrag der Floater zur präoperativen Visusminderung, wenn auch keine Signifikanz auf dem 5 %-Niveau erreicht wurde. Die Beeinträchtigung bei höhergradiger Fehlsichtigkeit ist nur mit dem Subindex zu Blendung (RBI2) lokal signifikant assoziiert. Denkbar ist, dass die höheren Beeinträchtigungswerte auch mit auf den Vernebelungseffekt durch die Fehlsichtigkeit zurückzuführen sind. Die postoperativen Beeinträchtigungswerte von Patienten, die 3 Monate nach Vitrektomie eine Indikation zur Kataraktoperation aufwiesen, waren vergleichsweise hoch (Abb. 2). Unter klinischen Alltagsbedingungen könnte dies für eine simultane Kataraktoperation mit der PPV sprechen.

Patienten mit Floatern am zweiten Auge berichteten höhere präop RBI, aber einen in etwa gleich hohen postop RBI wie Patienten ohne Floater am zweiten Auge (Abb. 3). Der Einfluss von Floatern am zweiten Auge manifestiert sich signifikant zum Subindex „RBI2 Blendung“. Möglicherweise erhöhen 2 mit Floatern belastete Augen die präoperative subjektive Blendung, weil am zweiten Auge die Störung am Studienauge in diesem Fall nicht kompensieren kann.

Je höher der präop RBI, umso höher der realisierte Rückgang der Beeinträchtigung und damit der Nutzen des operativen Eingriffs, der präop RBI kann damit als eine eigenständige prädiktive Größe herangezogen werden. Zu überlegen ist, zur Evaluation der subjektiven Beeinträchtigung im klinischen Alltag auf einen Floaterfragebogen zurückzugreifen. Soweit der für diese Studie entwickelte Fragebogen zur Anwendung kommt, können die in der Praxis erhobenen individuellen Daten mit denen in der Primärveröffentlichung und denen in dieser Auswertung publizierten im Sinne einer Einstufung des Schweregrades der subjektiven Beeinträchtigung abgeglichen werden.

### Treffsicherheit des augenärztlichen Befundes

Die in der Literatur berichtete vergleichsweise geringe Übereinstimmung von Untersucherbefund und subjektiver Einschätzung bestätigt sich in dieser Auswertung [6, 10]. Zwar weisen die Skalen eine schwach gleich gerichtete Korrelation auf – je größer laut Untersucher das Ausmaß der Floater im Studienauge, umso höher war der Median des präop RBI in der gesamten Population und in der Population von augengesunden Probanden –, allerdings waren die Abstände zwischen den präop RBI zu einigen der Untersucherklassen lediglich gering. Es wäre zu erwarten, dass der präop RBI bei lediglich „geringem Ausmaß von Floatern“ stärker von dem bei „erheblichem Ausmaß“ abweicht. Lediglich zu der Kategorie „massives Ausmaß von Floatern“ zeigte sich ein im Vergleich zu den anderen Kategorien deutlich höherer präop RBI. Etwas treffsicherer ist die Einschätzung des Behandlers in Patientenpopulationen, die mit Ausnahme von Floatern augengesund sind.

Die für die Studie entwickelte Skala der Untersuchereinschätzung zum Ausmaß der Floater verlangt eine qualitative Einstufung. Die geringe Assoziation legt nahe, dass die Skala von den Behandlern nicht übereinstimmend interpretiert und angewendet wurde. Möglicherweise sind die Kategorien „gering“, „mäßig“, „erheblich“ und „massiv“ nicht deutlich genug voneinander abgegrenzt. Für eine evtl. Folgeerhebungen schlagen wir eine Dreiteilung der Kategorien mit „geringem“, „mittlerem“ (hier fließen die verwendeten Kategorien mäßig und erheblich ein) und „massivem“ Ausmaß von Floatern vor.

Insgesamt bestätigt sich, dass Augenärzte bei der Beurteilung des subjektiven Beeinträchtigungsgefühls von Patienten durch Floater auch von deren subjektiven Einschätzungen profitieren können. Die Vorgehensweise, bei der Beratung soziodemografische und ophthalmologische Merkmale mit zu berücksichtigen oder ggf. einen Floaterfragebogen zur Quantifizierung der subjektiven Beeinträchtigung einzusetzen, scheint vor diesem Hintergrund adäquat.

### Methodische Gesichtspunkte

Mit der Auswertung konnten zwar zahlreiche Trends herausgearbeitet und z. T. mit lokaler Signifikanz in der univariaten Analyse unterlegt werden, insgesamt sind die Ergebnisse aber wenig belastbar. Insbesondere die multivariate lineare Regression lässt keine statistisch signifikanten Beziehungen zwischen den geprüften Merkmalen und dem präop RBI erkennen. Eine Ursache dürfte die geringe Fallzahl sein. Denkbar ist aber auch, dass die Abhängigkeiten zwischen Merkmalen und subjektivem Beeinträchtigungsgefühl tatsächlich eher schwach sind. Eine Überprüfung im Rahmen einer fallzahlstärkeren Untersuchung oder einer Metaanalyse wäre wünschenswert.

Die Studienpopulation setzt sich ausschließlich aus operationswilligen Floaterpatienten zusammen. Inwieweit sich die Ergebnisse in einem unselektierten Patientengut reproduzieren lassen, muss Gegenstand weiterführender Untersuchungen sein. Der Selektionsbias aufgrund der Qualifizierung der Patienten ist nicht einschätzbar. Allerdings stellt sich die Notwendigkeit für den behandelnden Arzt, auf Basis von soziodemografischen oder anderen ophthalmologischen Merkmalen als Floater eine Typisierung vorzunehmen, auch nur in der Patientenpopulation, deren Leidensdruck hoch genug ist, um auf eine Operation zu bestehen.

## Fazit

Die explorative Auswertung der Daten einer mit anderer Fragestellung befassten Primärstudie dient dazu, patientenbezogene Merkmale zu identifizieren, die bei operationswilligen Patienten mit Floatern mit höherer präoperativer subjektiver Beeinträchtigung und/oder niedrigerer postoperativer Beeinträchtigung nach Vitrektomie verbunden sind. Ziel der Studie ist es, dem Augenarzt für die Beratung eine Hilfestellung an die Hand zu geben. Dies scheint sinnvoll, da sich das Ausmaß der subjektiven Beeinträchtigung von Floaterpatienten und damit die Notwendigkeit einer Operation mit ophthalmologischen Standarduntersuchungen nur unzureichend erfassen lässt, zugleich aber immer wieder Patienten von sehr hohem Leidensdruck berichten. Die in dieser Studie von den Studienärzten verwendete Skala zu einer semiquantitativen Einschätzung des Ausmaßes von Floatern auf dem Studienauge konnte dieses Dilemma nicht beseitigen. Wie auch anderweitig in der Literatur berichtet, sind die Einschätzung des Behandlers und das subjektive Beeinträchtigungsgefühl der Patienten nicht kongruent.

In der Primärveröffentlichung konnte gezeigt werden, dass die präoperative subjektive Beeinträchtigung aufgrund von Floatern bei nahezu allen Patienten infolge der Vitrektomie massiv zurückgegangen ist. Dies galt selbst dann, wenn im Behandlungsverlauf eine intra- oder postoperative Komplikation auftrat. In dieser Analyse konnte das globale Ergebnis der Primärstudie im Sinne einer Typisierung von Floaterpatienten nach dem Ausmaß des subjektiven Beeinträchtigungsgefühls in Abhängigkeit von patientenbezogenen Merkmalen weiter differenziert werden. Das soziodemografische Merkmal, das mit vergleichsweise höchstem präoperativem einhergeht, ist Berufstätigkeit. Die Altersgruppe der 50- bis 70-Jährigen hat im Vergleich zu anderen Altersgruppen den größten Nutzen aus einer Vitrektomie. Jüngere Patienten erlebten trotz hoher Ausgangsbelastung eine etwas unterdurchschnittliche Reduktion der Beeinträchtigung nach Vitrektomie. Ältere berichten eine niedrige Ausgangsbeeinträchtigung, möglicherweise können sie sich nach Ausscheiden aus dem Berufsleben aufgrund anderer Anforderungen an das Sehvermögen eher mit Floatern arrangieren. Patienten mit einer weiteren ophthalmologischen Komorbidität – höhere Fehlsichtigkeit, Veränderungen der Netzhaut, niedrigerer Visus cc – berichteten über höhere Beeinträchtigungen zum Basiszeitpunkt, während die Beeinträchtigungswerte zum Endzeitpunkt 3 Monate nach Vitrektomie auf gleichem Niveau lagen wie bei den nicht belasteten Subgruppen. Es liegt die Vermutung nahe, dass Floater von Patienten mit zusätzlichen ophthalmologischen Krankheitsbildern weniger toleriert werden als von ansonsten Augengesunden und sie überproportional von einer chirurgischen Entfernung von Floatern profitieren. Liegt eine postoperative Trübung der Linse vor, ist der Nutzen einer isolierten Vitrektomie vergleichsweise geringer; dies spricht für eine simultane Kataraktoperation.

Für die Mehrzahl der Merkmale lässt sich weder über lokale Signifikanz noch über eine lineare Regressionsanalyse der Zusammenhang zu den Beeinträchtigungsindizes bestätigen. Möglicherweise wären dazu Studien mit höherer Fallzahl erforderlich. Denkbar ist jedoch auch, dass die Zusammenhänge nicht „hart“ sind, dass sich also das subjektive Beeinträchtigungsgefühl von Floaterpatienten aus mehr Faktoren und Merkmalen speist, als mit dieser Auswertung erfasst wurden. Mit Blick auf die betrachteten soziodemografischen und ophthalmologischen Merkmale bietet die Analyse jedoch ein besseres Verständnis, welche tendenziell mit höherer subjektiver Beeinträchtigung und größerem Nutzen aus einer Therapie vergesellschaftet sind.

## Fazit für die Praxis

Einige soziodemografische und ophthalmologische Merkmale von Floaterpatienten sind tendenziell mit höherer subjektiver Beeinträchtigung assoziiert: Berufstätigkeit, niedrigeres Alter, Floater am zweiten Auge sowie niedriger Visus, Veränderungen der Netzhaut und höhere Fehlsichtigkeit auf dem Studienauge. Niedrigere Beeinträchtigungswerte wiesen Patienten höheren Alters und ohne Erwerbstätigkeit auf. Je höher die präoperative subjektive Beeinträchtigung, umso größer ist der Gewinn einer Operation. Ein signifikanter „harter“ Zusammenhang lässt sich jedoch nicht regelhaft zeigen. Das vom Untersucher beurteilte Ausmaß von Floatern auf dem zu operierenden Auge ist ein unzureichender Prädiktor für die subjektive Beeinträchtigung. Zur Quantifizierung der subjektiven Beeinträchtigung in der Beratungspraxis sollte der Einsatz eines Floaterfragebogens erwogen werden.

## Abkürzungen

PPV: Pars-plana-Vitrektomie
RBI: Relativer Beeinträchtigungsindex
SA: Studienauge
SEQ: Sphärisches Äquivalent
VQoL: Visual Quality of Life Questionnaire
ZA: Zweites Auge

## Footnotes

1 Der Floater-Fragebogen wird auf Anfrage zur Verfügung gestellt. Bitte kontaktieren Sie Ursula Hahn (ursula.hahn@uni-wh.de).
